# Co-Existing Fungi: An Unforeseen Combo Creating a Dilemma in Diagnostic Morale

**DOI:** 10.7759/cureus.67600

**Published:** 2024-08-23

**Authors:** Lokesh Devalla, Babaji Ghewade, Pankaj Wagh, Vivek D Alone, Srinivasulareddy Annareddy

**Affiliations:** 1 Respiratory Medicine, Jawaharlal Nehru Medical College, Datta Meghe Institute of Medical Sciences, Wardha, IND

**Keywords:** histopathology, lung biopsy, cough, spore inhalation, immunosuppression

## Abstract

Mucormycosis is a rare fungal infection belonging to Mucorales order fungi. Rhino-cerebral form is more noted in patients with diabetes mellitus, but pulmonary mucormycosis is a rare manifestation with hematological malignancy, malignant tumors, and transplant recipient patients. Aspergillus species are also known to cause allergic bronchopulmonary aspergillosis, aspergilloma, chronic pulmonary aspergillosis, and invasive aspergillosis in immunocompromised individuals. We report a case of pulmonary mucormycosis superimposed with aspergillus infection presenting in an immunocompromised patient with metastatic prostate cancer on chemotherapy, initially misdiagnosed as aspergillus infection.

## Introduction

Mucormycosis, also known as black fungus, is a serious, rare fungal infection caused by a group of molds called mucormycosis, Rhizopus, and Mucor species. These environmental fungi mainly affect people with underlying immunosuppression. Inhalation of spores through the nose or mouth or even through a skin laceration mainly causes the infection. Immunocompromised patients may have inadequate cellular and humoral defense mechanisms, thus manifesting as devastating rhino-orbital, rhino-cerebral, and pulmonary infections [1]. However, any organ could get affected [2], and thus mucormycosis is termed cutaneous, rhino-cerebral, pulmonary, gastrointestinal, and disseminated [3] mucormycosis. Identification of non-septate hyphae in tissue on histopathology on culture [4] confirms the diagnosis of mucormycosis. Management involves a combination of antifungal therapy and aggressive surgical debridement of involved tissues [5,6].

## Case presentation

**Figure 1:**
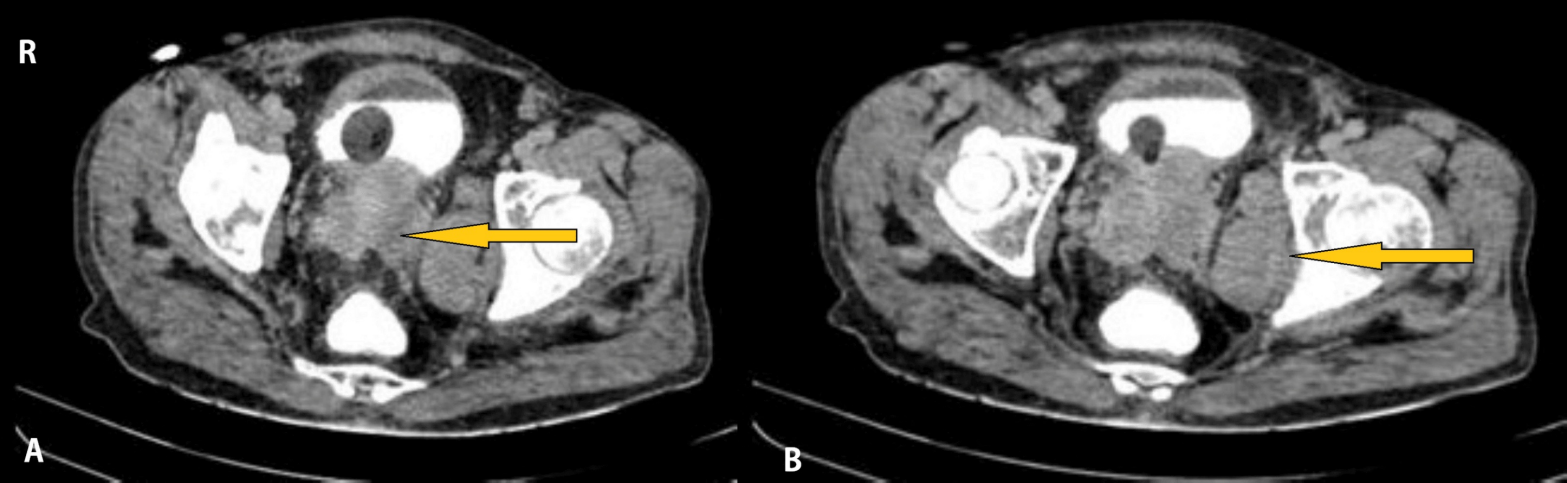
Contrast CT of the abdomen and pelvis showing (A) heterogeneously enlarged prostate with invasion of the surrounding tissue and (B) enhancing deposits in the left internal iliac region and left obturator muscle.

A 48-year-old man presented with non-radiating chest pain for eight days, dyspnea, and cough with white mucoid expectoration. He had a history of difficulty in urination with increased frequency and dribbling of urine, along with symptoms of weight loss and anorexia. He was also a known case of prostate carcinoma with multiple metastases and is currently on docetaxel chemotherapy. Contrast CT of the abdomen and pelvis are shown in Figure 1.

His lab parameters were normal. Assuming pulmonary metastasis, CT of the thorax was performed, which revealed ground-glass opacities in the right upper lobe with no pleural effusion and normal left lobes. Upon bronchoscopy, the right-sided upper lobe bronchus revealed thick mucoid secretions. Bronchoalveolar lavage (BAL) was sent for culture, which revealed growth of fungal elements positive for periodic acid Schiff and hexamine silver stain with septate hyphae with acute angled branching. Diagnosis of probable invasive pulmonary aspergillosis was made, and he was started on oral voriconazole for 30 days, showing improvement in further scans, and thus he was discharged with follow-up.

**Figure 2:**
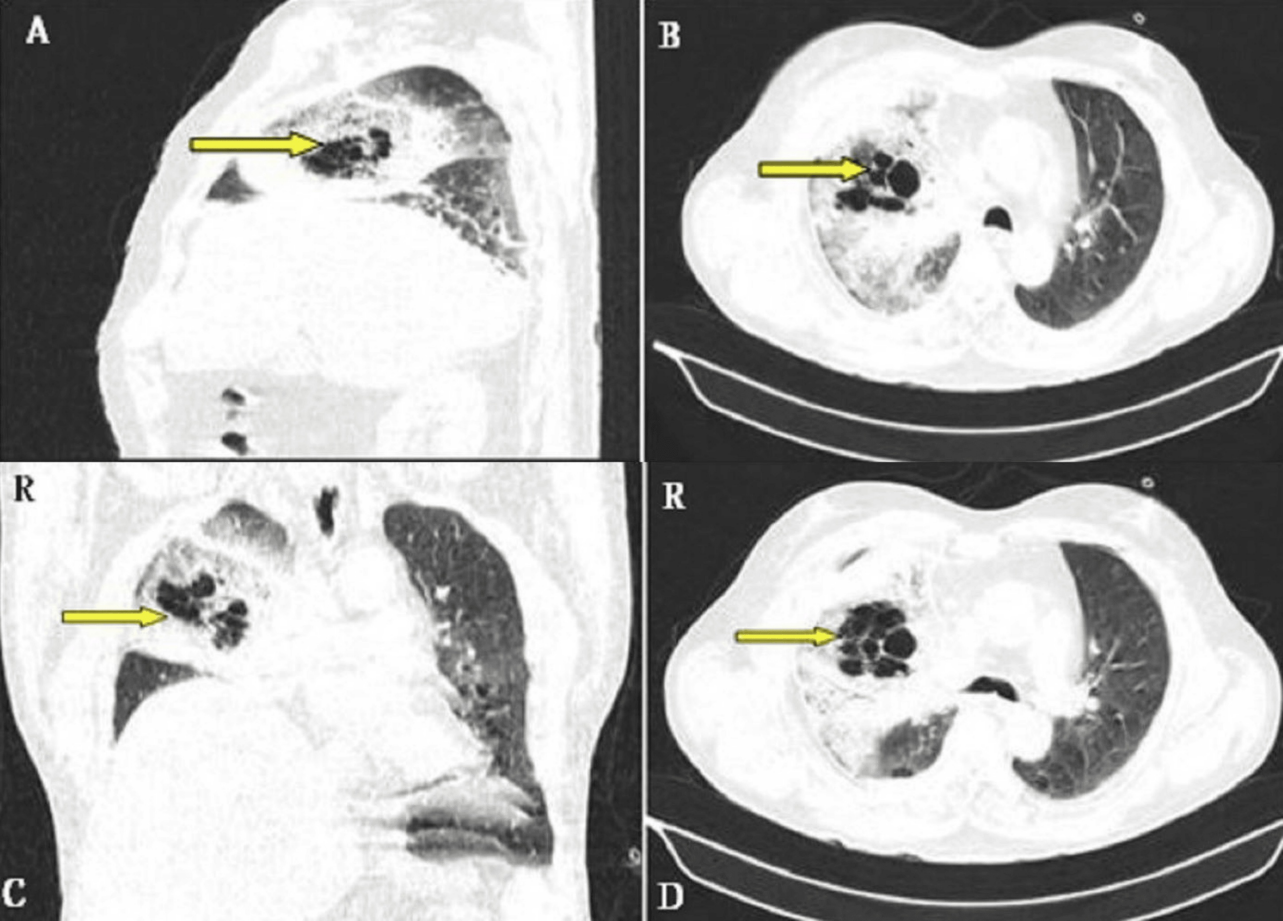
High-resolution computed tomography of the thorax showing multiple cystic cavitations with surrounding consolidation in a nest pattern described as a bird's nest sign (yellow arrows).

After a month, he presented with severe breathlessness and increased cough with no reduction in symptoms. Chest X-ray showed increased right upper zone haziness. CT of the thorax was repeated, and this time it suggested the presence of conglomerated bubbly cystic lucencies in the right upper lobe known as bird's nest appearance, significant surrounding consolidation, and ground-glass attenuation with interlobular septal thickening involving almost the entire right upper and middle lobes as well as a thin strip of multifocal right-sided pleural effusion (Figure 2).

**Figure 3:**
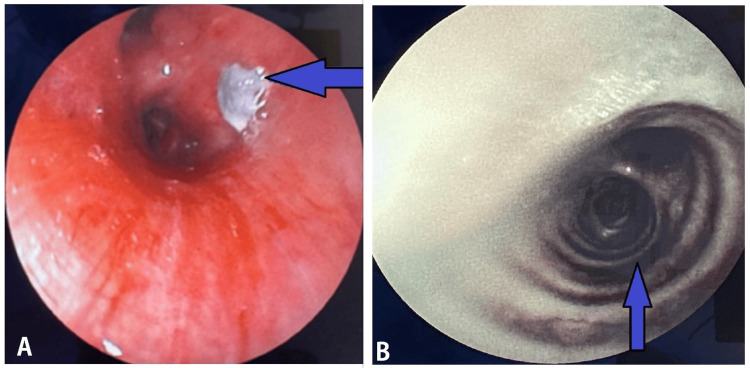
FOB showing fungal elements along with black right upper lobe bronchus path (blue arrows). FOB, fiberoptic bronchoscopy

Blood investigations showed significant neutropenia of 300 cells per microliter of blood. Peripheral smear showed normocytic hypochromic red blood cells with leukopenia and adequate number of platelets. Repeat bronchoscopy of the right-sided upper lobe bronchus revealed a blackish mass along with proceeding necrotic bronchial airways (Figure 3).

**Figure 4:**
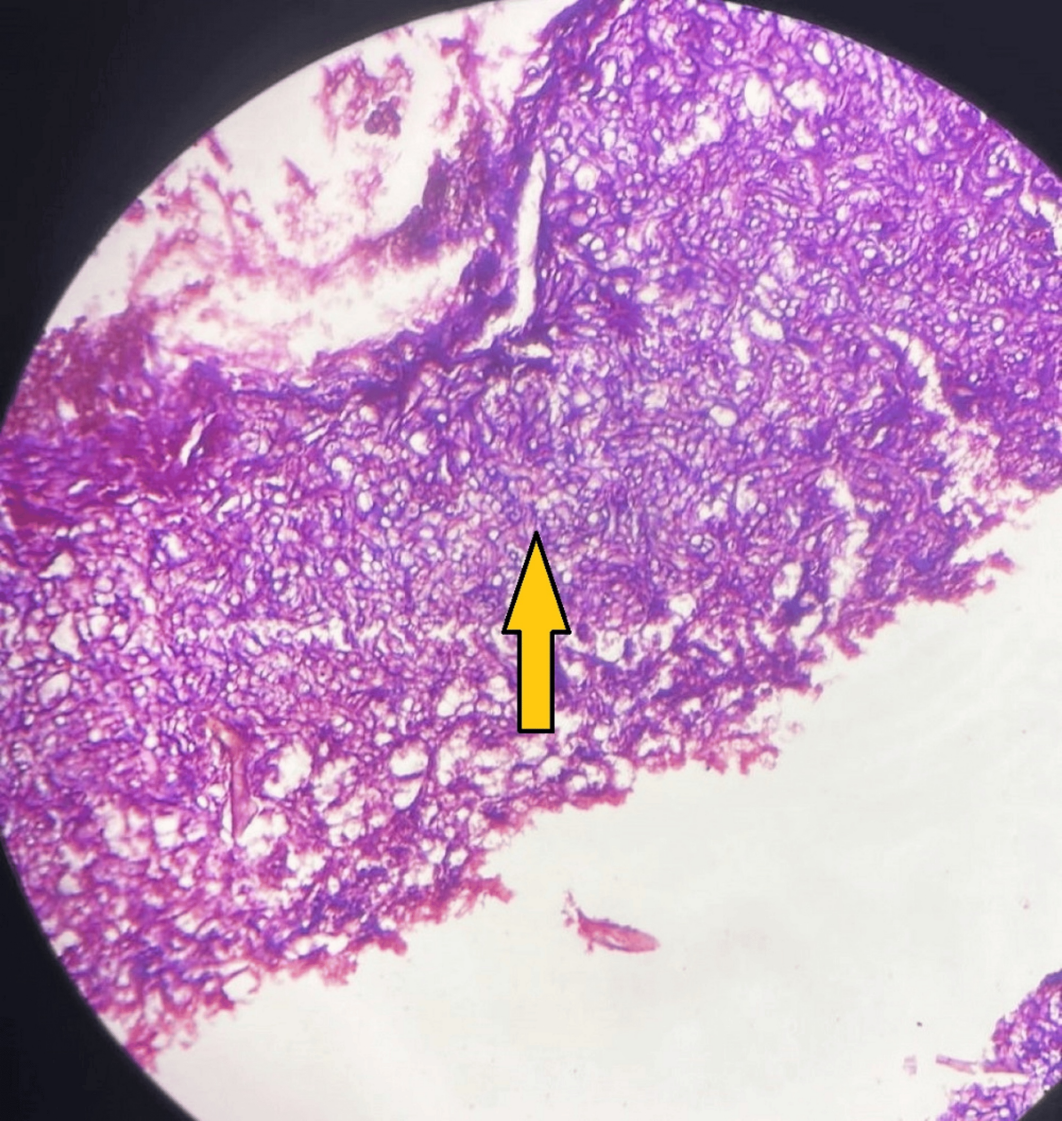
Microscopy from HPE of TBLB specimen showing aseptate hyphae at right angles, suggesting mucormycosis, as indicated by a yellow arrow. HPE, histopathological examination; TBLB, transbronchial lung biopsy

Transbronchial lung biopsy (TBLB) was performed, and histopathological specimen revealed fungal elements, which, on microscopy, showed broad aseptate ribbon-like hyphae with right-angled branching (Figure 4).

This investigation confirmed the presence of mucormycosis but did not show Aspergillus fungal elements. This was assumed to be due to the superimposed infection of mucormycosis and Aspergillus, where the latter was controlled by voriconazole. Immediately, liposomal amphotericin B was started intravenously. The patient gradually improved clinically, but radiological scans persisted the same, and thus he was transferred to the surgical department for lung resection procedures.

## Discussion

Pulmonary mucormycosis is a rapidly progressive infection transmitted by the inhalation of spores. Spores are larger than those of *Aspergillus fumigatus* [7], the common organism implicated in pulmonary aspergillosis [8], commonly entrapped in the upper airway and sinuses. However, occasionally, they can bypass the upper airway into the lower airway, leading to clinical symptoms of fever, dry cough, shortness of breath, chest pain, and hemoptysis [1,3]. It does not generally coexist with Aspergillus but should be ruled out in fungal infections not resolving with treatment.

The standard diagnosis of mucormycosis is the isolation of the organisms in the tissues with culture confirmation. However, the positivity rate of cultures is very low, and evidence of infection is a histopathologic identification of an organism, typical of Mucorales. Also, high chances of contamination interfere with the evidence of this fungus in culture. Microscopic evidence for pulmonary mucormycosis is usually obtained from sputum or BAL specimens. TBLB showing broad, non-septate hyphae, branching more irregularly at acute angles, is characteristic. Diagnosing pulmonary mucormycosis is difficult because of the non-availability of tissue for the demonstration of the organism and the similarity of clinical presentation with other invasive fungal infections. Therefore, radiographic evidence (chest X-ray, CT) is often used to support the diagnosis [1].

CT showing a "bird's nest sign" describes the appearance of a reverse halo sign in combination with erratic lines of consolidation or stranding zones located around areas of ground-glass opacity [9]. Bird's nest and reverse halo are not only restricted to invasive fungal disease, but they can also appear in other conditions such as cryptogenic organizing pneumonia, bacterial pneumonia, paracoccidioidomycosis, sarcoidosis, Wegener granulomatosis, and tuberculosis [9]. It is critical to differentiate between mucormycosis and aspergillosis due to the potential differences in treatment (voriconazole is ineffective against mucormycosis) and because appropriate early treatment of mucormycosis may improve the outcome of the patient. In a diabetic and neutropenic patient [9-12], imaging signs such as the bird's nest sign, lung nodules of more than 10, pleural effusion, concurrent sinusitis, and degrading patient status despite voriconazole prophylaxis are more characteristic of mucormycosis than Aspergillus infection. Both fungi grow together masking the other. Difficult diagnosis is resolved by noninvasive procedures such as transbronchial lung biopsy and histopathology.

Invasive mucormycosis is implicated to be associated with underlying pathological conditions such as diabetes mellitus (especially ketoacidosis state), glucocorticoid therapy, malignancies, hematopoietic cell transplantation, and solid organ transplantation. Other risk factors are the use of deferoxamine, iron overload, AIDS, trauma/burns, and malnutrition. Voriconazole prophylaxis is found to be an independent risk factor in post-transplant patients, but the reason is not clear [1]. No identifiable risk factors [3] are noted in some conditions. Rhizopus species, one of the major implicated organisms in mucormycosis, thrive in ketoacidosis conditions as they possess an enzyme called ketone reductase that makes them capable of utilizing ketones as an energy source [1]. Mucormycosis is seen commonly in patients who underwent iron chelation as they prefer a high iron environment and deferoxamine-iron chelate (Feroxamine), which acts as a siderophore and increases the iron uptake by the fungus. Feroxamine abolishes the fungistatic effect of serum on Rhizopus and increases the growth of the fungus [13]. Statins are believed to have activity against a wide range of agents of mucormycosis [1].

Management of mucormycosis involves a combination of surgical and pharmacological therapy. Elimination or treatment of underlying immunosuppression is necessary. Aggressive surgical debridement of involved tissues should be considered. Intravenous (IV) amphotericin B (lipid formulation), started at 5 mg/kg daily and increased to 10 mg/kg daily, is the drug of choice for initial therapy. Early initiation of antifungal therapy improves the outcome of infection with mucormycosis [1]. After the patient responds, posaconazole or isavuconazole can be used for oral step-down therapy. Amphotericin B should be continued until the patient has shown signs of improvement, which will usually take several weeks. In patients who do not respond to amphotericin B, posaconazole or isavuconazole is used as an alternative. Other antifungal agents, including voriconazole, fluconazole, and flucytosine, are not effective against the Mucorales. Although the echinocandins are not used in clinical practice to treat mucormycosis, they could be useful as *Rhizopus oryzae*, the most common cause of mucormycosis, expresses the target enzyme for echinocandins. Combination therapy is not recommended. Pharmacological therapy continues till there is clinical and radiographic resolution of active disease. It usually takes months, and some patients remain on therapy for life [1].

## Conclusions

Invasive mucormycosis is a rare but aggressive fungal infection with high morbidity and mortality, particularly in patients with underlying medical comorbidities or immunosuppression. Clinical radiographical and histopathological presentations can vary, mimicking or coexisting with aspergillus infections between patients based on the immune status of the host and mode of infection. However, it is important to rule out other fungal infections confusing treatment protocols and keep a high level of suspicion, as early diagnosis and rapid initiation of surgical and antifungal therapy are key to improving survival.
